# From Hemp to CBD Crystals: A Scaled-Up Procedure for the Selective Extraction, Isolation, and Purification of Cannabidiol

**DOI:** 10.1021/acsagscitech.4c00462

**Published:** 2025-02-17

**Authors:** Roberto Calmanti, Maurizio Selva, Alvise Perosa

**Affiliations:** Dipartimento di Scienze Molecolari e Nanosistemi, Università Ca’ Foscari Venezia, Via Torino 155, Venezia 30172, Mestre, Italy

**Keywords:** cannabidiol, hemp, CBD isolation, supercritical CO_2_ extraction, THC-free

## Abstract

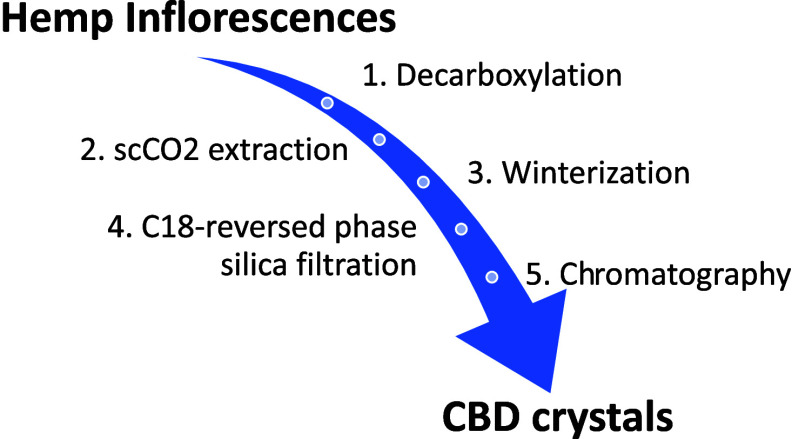

The isolation of cannabidiol (CBD) with a purity greater than 99%, while avoiding the presence of Δ9-tetrahydrocannabinol (THC), is essential for both exploring CBD applications and its commercialization. The scientific literature lacks robust and scalable protocols for obtaining pure CBD, instead primarily focusing on obtaining hemp extracts with higher cannabinoid content. Herein, we present a complete procedure for obtaining pure CBD crystals, starting from an industrial hemp cultivar. The protocol includes thermal decarboxylation of cannabinoids, supercritical CO_2_ extraction, winterization of the extract, CBD purification via sequential C18 reverse-phase silica filtration, silica gel chromatography, and selective crystallization. Each step has been carefully optimized to identify the best solvent, solvent/extract ratio, and silica/extract ratio to minimize CBD loss in waste fractions and to enable solvent and material recovery for recycling, aligning with a sustainable perspective. The scale-up of the procedure to 100–600 g for each step demonstrated the feasibility of our protocol for the obtainment of pure CBD crystals. An overall 52% yield of CBD with a purity exceeding 99% and a negligible THC content was achieved.

## 1 Introduction

Cannabis has intertwined with human history since ancient times, with hemp fiber use dating back around 12,000 years in China and medical cannabis records from 5000 BC in central Europe.^1^ Its versatile applications include nutrition, paper and plastic production, textiles, insulation, and various pharmacological and cultural uses.^2,3^

The chemistry of cannabis has been quite extensively studied, identifying approximately 500 compounds such as terpenoids, flavonoids, hydrocarbons, sugars, and nitrogenous compounds.^4,5^ The recent scientific research is focusing on the constituents contained in the resin secreted by the head cells of granular hairs (trichomes) distributed across the surface of cannabinoid plants, in particular cannabinoids.^6^ The pharmacological activity of cannabis has been attributed to these compounds as they have been shown to interact with the ECS (endogenous cannabinoid system).The discovery of this system was undoubtedly facilitated by the prior isolation and elucidation of the structure of the phytocannabinoids found in cannabis.^7^

Out of approximately 150 cannabinoids (CBNDs) identified in *Cannabis sativa*,^8^ 90 have been purified and fully characterized.^8^ Among these, the most common are cannabidiol (CBD), Δ9-tetrahydrocannabinol (Δ9-THC), cannabigerol (CBG), cannabinol (CBN), cannabichromene (CBC), tetrahydrocannabivarin (THCV), and cannabicyclol (CBL), which usually represent 10–30% w/w of inflorescences on a dry basis.^9^ These compounds are usually found in their acidic form (e.g., CBDA, Δ9-THCA, CBGA, CBNA, etc.) containing a carboxylic acid moiety, which are the precursors of the decarboxylated species. The relative abundances of the main CBNDs strongly depend on various factors such as the chemical phenotype of the cultivated variety, the maturity of the plants at the harvest related to the biosynthetic pathway of formation of each CBND from precursors during the plant development, the storage conditions, the soil, the climate, the time of collection, and geographical location.^10^

CBD has gained significant importance in the last 20 years, as evidenced by scientific publications on the subject. The number of publications discussing CBD and its applications increased from 25 in 2002 to 1082 in 2021, according to the ISI’s Web of Knowledge database. This surge is due to the disclosure of its ability to treat various disorders such as anxiety, pain and inflammation, multiple sclerosis, etc., coupled with its nonpsychotropic nature (unlike Δ9-THC).^11^ It seems to be a new “miracle” cure, even though some uncertainties remained on the quality and purity of commercialized CBD oils and extracts because of the growing demand.^12^

Many scientific articles and reviews have explored the potential for extracting cannabinoids from fresh or dried plant material.^9,13^ The classical methods for the extraction of natural compounds are conventional solid–liquid procedures involving maceration, percolation, or Soxhlet extraction with organic solvents.^14^ Recently, novel and greener extraction techniques have been reported by using supercritical carbon dioxide (scCO_2_), deep eutectic solvents and ionic liquids, ultrasound- or microwave-assisted procedures, hard-cap espresso machines that exploit pressurized hot water, etc.^15^ Although initial costs are higher compared to a conventional procedure based on organic solvents, the use of scCO_2_ for natural compound extraction is increasingly garnering attention in the pharmaceutical and food industries due to its safety and nontoxicity. Numerous scientific studies are published regarding the extraction of CBNDs through scCO_2_.^16−27^

The procedures mentioned so far were limited to the obtainment of cannabis extracts containing CBNDs, with few investigations reported on the separation of CBD and THC.^28^ This scarcity indicates the significant challenge in purifying these compounds due to their close structural similarity. On the other hand, the separation of CBD and THC is essential for the commercialization of hemp extracts since, in many countries, THC is a controlled substance, and its content in commercial products must be <0.2%,^29^ while the availability of pure cannabinoids is essential for numerous medical applications.^30^ Our investigation aims to develop a simple and efficient route to obtain CBD with a purity greater than 99% and a THC content below 0.2%. There is a significant lack of such procedures, particularly scalable and reproducible processes that can be used to explore possible CBD applications but also to produce commercial formulations.^31^

Various studies have focused on the procedure for the isolation of THCA: Lehmann and Brenneisen extracted the plant material with acidified petroleum ether (PE), followed by two subsequent solvent extractions with basic aqueous solution and diethyl ether. The extract as obtained was then submitted two times to medium pressure liquid chromatography on a reversed-phase (RP) silica, yielding 50 mg of THCA from 50 g of initial extract.^32^ Dussy et al. reported a similar method using a manually packed silica column with an elution solvent system mixture of toluene, hexane, acetone, and acetic acid.^33^ Wohlfarth et al. reported another method based on a first ethanol extraction followed by a normal phase silica column (120 g of silica for 1.8 g of extract) with cyclohexane and acetone modified with pyridine as a gradient mobile phase.^34^ Finally, some researchers reported the use of partitional chromatography using a two-phase system and eventually the pH-zone refining method to obtain CBD and THC on a preparative scale.^6,35^

As for CBD, industrial production is mainly pursued through short-path or wiped-film distillation, but a significant amount of CBD is discarded in the waste fractions of the process without any chance to recover it.^36^ Scientific papers reported lab-scale procedures to obtain pure cannabidiol or other cannabinoids in order to test it in structural pharmacological activity studies, but since their scope is different, the yields in cannabinoids are usually very low (0.5–2.5% with respect to the initial extract).^37−42^ The obtainment of CBD with a purity >94% was also reported through innovative techniques such as two-dimensional liquid chromatography and fast centrifugal partition chromatography, but they cannot be considered scaled-up procedures.^43,44^

Recently, Marzorati et al. reported an interesting option in which hemp is extracted through scCO_2_ followed by winterization and flash chromatography to obtain a CBD-rich oily extract (>80% w/w), but no crystallization and/or complete purification of CBD was reported.^28^ Olejar et al. reported another route that exploits dynamic maceration, a pressurized ethanol liquid extraction/decarboxylation step followed by a C18-reversed-phase chromatography to isolate CBD with a purity of 91.8%.^45^ Some patents were also registered on the isolation of CBD by solvent extraction followed by chromatography,^46−50^ use of adsorbent resins,^51^ molecular distillation,^52−55^ or alkali extraction followed by reactive crystallization.^56−58^

**Figure 1:**
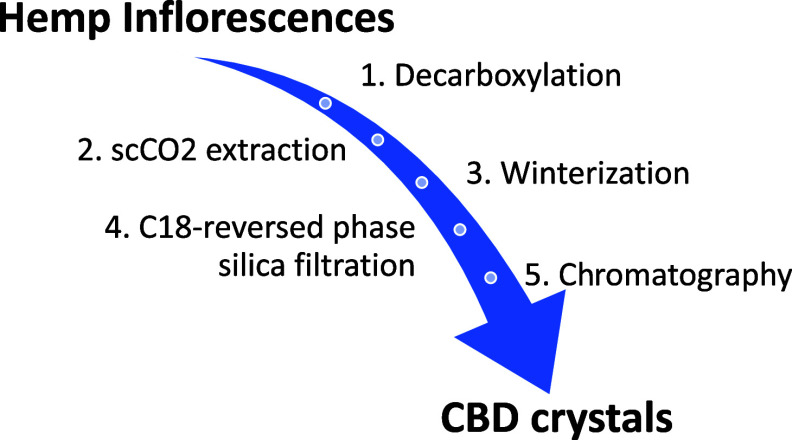
Schematic representation of our protocol for the obtainment of CBD crystals from hemp inflorescences.

In this work, we present a novel procedure to selectively extract CBD from an Italian *Cannabis sativa* cultivar with a CBD content of approximately 5% w/w in its inflorescences (see Figure 1 for a process overview). Our study provides a detailed investigation of every step required to achieve high-purity CBD extraction.

Starting from decarboxylation and scCO_2_ extraction, we conducted a thorough analysis of the optimal solvents, extract/solvent ratios, and extract/silica ratios for each purification step, namely, winterization, C18-reversed-phase filtration, and chromatography. Following laboratory-scale optimization (10–50 g), these steps were successfully scaled up using 100–600 g of starting material, demonstrating the scalability of the process. We also prioritized the recovery and recyclability of solvents and materials to improve the sustainability of the procedure.

While decarboxylation and scCO_2_ extraction have been well-documented in previous studies,^16−28,45,59−62^ there remains a significant gap in the literature regarding the optimization of conditions for winterization, C18-RP silica filtration, chromatography, and recrystallization. Our study fills this gap by rigorously optimizing each step for the large-scale production of CBD with a purity exceeding 99%. This comprehensive approach not only benefits research in cannabinoid extraction but also holds potential for commercial applications, offering a scalable and efficient method for extracting cannabinoids and other natural compounds from hemp.

## 2 Materials and Methods

The hemp cultivar *Enectaliana* was grown in Italy, and its milled inflorescences (granulometry < 1.5 mm) were kindly provided by Enecta SRL, Bologna, Italy. The amounts of CBD, CBDA, THC, and THCA in pristine hemp were determined using the technique described in Section 2.1. The quantification was further validated by high-performance liquid chromatography (HPLC) analysis conducted by an external laboratory (Istituto Zooprofilattico Sperimentale del Mezzogiorno, Portici, Italy), as detailed in the Supporting Information section.

Anthracene, acetonitrile, ethanol, ethyl acetate, *n*-hexane, and petroleum ether were purchased from Sigma-Aldrich. Liquid carbon dioxide was SFC grade and supplied by SIAD SpA.

Silica gel 60 and C18 reversed-phase silica were purchased by Sanpont Group, Yucheng Chemical, and Shanghai Co., Ltd.

^1^H NMR and ^13^C NMR were recorded on a Bruker NMR spectrometer (400 MHz for ^1^H NMR; 100 MHz for ^13^C NMR). Chemical shifts were reported in δ values downfield from TMS; deuterated chloroform and methanol were used as the deuterated solvents. GC–MS (EI, 70 eV, Agilent Technologies, Santa Clara, CA, USA) analysis was performed on an HP5-MS capillary column (*L* = 30 m, Ø = 0.32 mm, film = 0.25 μm) purchased from Agilent Technologies (Santa Clara, CA, USA). The following conditions were used: carrier gas: He; flow rate: 1.0 mL/min; split ratio: 10:1; initial T: 50 °C (2 min); ramp rate: 15 °C/min; and final T: 300 °C.

### 2.1 Determination of CBD, CBDA, THC, and THCA through the ^1^H NMR ERETIC Technique

The ^1^H NMR ERETIC (electron reference to access in vivo concentration) technique was the external calibration method used to quantify the amount of CBD/CBDA and THC/THCA in hemp samples and in the cannabinoid-containing extracts by using anthracene as a standard.^63^ The identification peaks used for the quantification were, respectively, the signals at δ = 5.57, 4.66, and 4.56 ppm for CBD and 6.14 ppm for Δ9-THC, as already reported in previous papers on the quantitative analysis of cannabinoids by ^1^H NMR.^64,65^ The ERETIC method was calibrated weekly, and the accuracy to standard was ±3%. All quantifications of CBD and THC in hemp and extract samples were duplicated, with the values determined by ^1^H NMR differing by less than 3% between experiments.

#### 2.1.1 Determination of the Amount of CBD, CBDA, THC, and THCA in Both the Pristine Hemp and the Residual Hemp after Extraction

The quantification of cannabinoids in the dried milled hemp inflorescences was conducted by NMR, adapting a technique outlined by the United Nations Office on Drugs and Crime.^66^ Briefly, ∼100 mg of hemp was placed in a 10 mL glass tube, followed by the addition of 1 mL of a 9:1 CD_3_OD/CDCl_3_ solution. The sample was sonicated for 15 min followed by centrifugation (6000 rpm, 15 min). The supernatant was collected and quantitatively analyzed by NMR using the ERETIC technique.

#### 2.1.2 Determination of the Amount of CBD, CBDA, THC, and THCA in the Extracts Obtained after Each Step

A small amount of hemp extract (approximately 15 mg) was accurately weighed in an NMR tube, and 600 μL of deuterated chloroform was added to dissolve the extract. The solution was quantitatively analyzed by NMR using the ^1^H NMR ERETIC technique.

### 2.2 Decarboxylation of Hemp Inflorescences

Milled hemp inflorescences were spread on a glass sheet (70 cm ×100 cm) to achieve a plant material width of approximately 10 mm. The glass sheet was then placed in a laboratory oven maintained at 80 °C for 24 h to decarboxylate CBDA into CBD following the optimization described elsewhere.^60,66^ After decarboxylation, the amounts of CBD, CBDA, THC, and THCA were determined through ^1^H NMR analysis on hemp using the ^1^H NMR ERETIC technique.

### 2.3 Supercritical CO_2_ Extraction

The milled hemp inflorescences were extracted in a scCO_2_ extractor pilot plant (OL94 GREEN OIL, Berengo spa) managed by CSA—Università Ca’ Foscari di Venezia. A detailed description of the pilot plant is reported in Supporting Information and Figures S1 and S2.

In a typical procedure, ∼0.6 kg (5 L in uncompressed volume) of hemp was charged in the vessel and extracted with supercritical CO_2_ at a flow rate of 25 kg/h. The extractor vessel was kept at 250 bar and 40 °C for 3 h by using an expansion valve to control the pressure of the system. The gravimetric separator and the cyclonic separator S3 were kept at 50 bar and 35 °C, and the extract was collected at regular intervals, opening an automated valve for 10 s every 5 min. The extracts from the two separators were collected, combined, and analyzed by ^1^H NMR to quantify the cannabinoids. To do this, a small amount of hemp extract (approximately 15 mg) was accurately weighed in an NMR tube, and 600 μL of deuterated chloroform was added and quantitatively analyzed by NMR using the ^1^H NMR ERETIC technique. The presence of CBD in the residual hemp was tested using the same method described above for the pristine hemp.

The procedure was repeated several times to obtain the quantity of extract necessary for conducting all tests related to winterization, C18 reverse-phase silica filtration, and silica gel chromatography, which will be described in the following paragraphs. The extraction yield and CBD content are reported as the mean of five extractions.

### 2.4 Winterization

A round-bottomed flask equipped with a condenser and a stirring bar was charged with 20 g of sc-CO_2_ extract dissolved in the proper amount of the selected solvent (i.e., acetonitrile, ethanol, ethyl acetate, and *n*-hexane) with an extract/solvent ratio = 1:3–10 w/w. The solution was stirred for 1 h at 80 °C; after that, it was rapidly cooled to −18 °C in a freezer and kept at this temperature for 24 h. The solution was finally filtered and concentrated under reduced pressure (50 °C, 20 mbar) to recover the CBD-rich extract and recycle the solvent. ERETIC ^1^H NMR analyses were performed to quantify the amount of CBD contained both in the extracted CBD-rich fraction labeled dewaxed extract (DE) as well as in the waste, i.e., the solid waxy fraction (WF).

After optimization, the winterization step was performed on a larger scale using 100 g of scCO_2_ extract dissolved in 300 g of CH_3_CN (solvent/extract mass ratio = 3:1) to demonstrate the scalability of the process. In this case, the WF was redissolved in the appropriate amount of CH_3_CN (solvent/extract ratio = 3:1 w/w), and the winterization step was repeated twice to recover the maximum amount of CBD contained in the waxy fractions.

Data are expressed as mean percentage values obtained from at least two independent experiments, with a standard deviation of ±1.9% for the extract/starting material percentage ratio.

### 2.5 C18-Reversed-Phase (RP) Vacuum Filtration

The dewaxed extract obtained from the winterization step was used for the C18-RP filtration step. DE (20 g) was dissolved in the minimum amount of the selected solvent (∼10 mL) and placed directly on the stationary phase (C18-RP silica/extract = 3–10:1 w/w) previously dry-packed into a column (diameter 3.5 cm, length 10 cm) equipped with a vacuum connection. The filtration was performed by applying vacuum at the outlet with 500 mL of the selected solvent to yield a first purified CBD-rich fraction labeled as C18-extract. The C18-RP silica was subsequently washed and regenerated by elution of 300 mL of ethyl acetate (or tetrahydrofuran) in order to reuse it several times, and the second ethyl acetate fraction was labeled as C18-waste. Both fractions (C18-extract and C18-waste) were concentrated by a rotary evaporator (50 °C, 20 mbar), with solvents being recovered and reused multiple times. ERETIC ^1^H NMR analyses were conducted to quantify the amount of CBD contained in both the extract and waste fractions.

Following the optimization of the filtration process at a 20 g scale, the procedure was repeated using 150 g of dewaxed extract to demonstrate the scalability of the method. A dry-packed column (10 cm diameter and 80 cm length) equipped with a vacuum connection was charged with 450 g of C18 reverse-phase silica. The C18-extract was obtained by using 1.5 L of CH_3_CN. The C18-RP silica was regenerated by the subsequent elution of 2 L of ethyl acetate to remove the C18-waste fraction and enable the reuse of the silica.

### 2.6 Silica Gel Chromatography and Crystallization of CBD

The extract obtained from the C18-RP filtration step was subjected to liquid chromatography. 50 g of C18-extract was mixed with 10 g of silica gel and 20 mL of petroleum ether (PE). This homogeneous slurry was placed directly on the silica gel stationary phase (silica/extract = 7:1 w/w) previously wet-packed with PE in a glass column (diameter 4.5 cm, length 50 cm). The mobile phase consisted of a 9:1 PE/ethyl acetate mixture. A slight overpressure of air was imposed on the column head, sufficient to reach a constant mobile phase elution rate of 35 mL/min.

The fractions (each of 100 mL) were monitored by thin-layer chromatography (TLC) using a KMnO_4_ solution as a visualization reagent.

The appropriate fractions were combined and concentrated under reduced pressure to obtain a reddish oil, and CBD crystals were added to favor rapid crystallization. Once formed, the crystals were suction filtered and washed repeatedly with cold *n*-hexane. A further recrystallization was performed by dissolving crystals in the minimal amount of hot *n*-hexane and allowing them to recrystallize at room temperature. The CBD crystals were subjected to high vacuum (0.1 mbar) at 40 °C to eliminate any trace of solvents and finally analyzed by NMR (^1^H, ^13^C, COSY, HMBC, and HMQC; see Supporting Information Figures S3–S7) and GC–MS (Figures S13 and S14).

After optimization at a 10 g scale, the process was scaled up to 500 g of extract obtained from the C18-RP filtration step. The scaled-up process was adjusted to carry out silica purification by gravity elution using the apparatus depicted in Figure S11. In this case, 3.5 kg of silica was wet-packed with petroleum ether (PE) in a glass column (15 cm diameter and 150 cm length), and a 9:1 mixture of petroleum ether and ethyl acetate was employed as the mobile phase. Fractions of 800 mL were eluted, and those containing CBD were combined and concentrated under reduced pressure to obtain a reddish-brown oil (Figure S12a). From this oil, 183 g of yellowish CBD crystals (Figure S12b) was obtained.

The CBD crystals were redissolved in a minimal amount of hot *n*-hexane and allowed to recrystallize, yielding 155 g of pure CBD after high-vacuum treatment to eliminate any trace of solvents. The purity of the obtained CBD was confirmed by HR-GC-FID analysis conducted by an external laboratory (FOR.MED.LAB.—Forensic Medicine and Laboratory S.R.L., Macerata, Italy), with the analytical report provided in the Supporting Information.

## 3 Results and Discussion

The combined amount of CBD and CBDA present in the milled inflorescence was measured using ^1^H NMR and was found to be 4.8% w/w, whereas the quantity of THC + THCA was <0.2% w/w (see Figure S8). This finding was confirmed by HPLC analysis conducted by an external laboratory. (Istituto zooprofilattico del Mezzogiorno, Portici, Italy).

Milled inflorescences were used for the elaboration and the investigation of a multistep protocol, which is depicted in Figure 1 and separately outlined in the subsequent paragraphs.

### 3.1 Decarboxylation of Acidic Cannabinoids Contained in Hemp Inflorescences

Most authors agree on the importance of a thermal decarboxylation step to convert CBDA to CBD before scCO_2_ extraction as this enhances the solubility of cannabinoids compared to their acidic counterparts. Several previous studies have explored the optimal experimental conditions for the decarboxylation of CBNDs from different hemp strains, considering factors such as the presence of oxygen, the presence of solvents or sorbents, the amount of plant material, the cannabinoid profile, etc.^20,22,28,45,59−62^ Although a quick decarboxylation at high temperature may seem less energy-intensive, a recent and thorough kinetic study on the decarboxylation of CBNDs clearly demonstrates that the use of low temperature (*T* = 80–100 °C) and longer time (10–24 h) is preferable for maximizing the yield of CBD. In contrast, higher temperatures (*T* = 140–170 °C) and shorter times (*t* = 5–60 min) are better suited for maximizing the amount of other neutral CBNDs (e.g., THC and CBN), which are undesirable in our case.^60^ Given the lack of novelty regarding the investigation of decarboxylation conditions, we followed the optimization conducted by Moreno et al. by performing decarboxylation at 80 °C for 24 h in an air oven. This ensured complete conversion of CBDA to CBD, although a negligible loss was observed, as confirmed by ^1^H NMR analysis (CBD = 4.5 ± 0.1% w/w).

### 3.2 Supercritical CO_2_ Extraction of Decarboxylated Hemp Inflorescences

As reported in the Introduction part, a plethora of studies have recently been published regarding the extraction of cannabinoids with scCO_2_ technologies and the evaluation and optimization of parameters (temperature, CO_2_ pressure, extraction time, and cosolvent presence) for the extraction of high CBD or THC content from various hemp strains, as well as from industrial hemp residues.^16−27,67^ The majority of these publications employed temperatures between 37 and 50 °C, CO_2_ pressures ranging from 165 to 320 bar, extraction times of 3 to 10 h, and the addition of 2 to 10% v/v ethanol as a cosolvent. A comprehensive comparison of the conditions used is reported elsewhere.^21,68^ The inclusion of a cosolvent (i.e., ethanol) appears to enhance CBD extraction but also the overall yield due to its ability to coextract chlorophylls and other compounds from the starting material.^20,21,24,25^ However, our objective is to extract all of the CBD contained in the hemp without specifically optimizing the scCO_2_ extraction conditions as numerous valuable papers have already optimized these parameters and inspired our selection of experimental conditions for CBD extraction. The chosen temperature and pressure were justified through a careful analysis of recent reviews and research papers that advocate the use of 250 bar and 40 °C for cannabinoid extraction from hemp.^25,27,69^ The CO_2_ flow rate (25 kg/h) was adjusted based on the size of our pilot plant and aligned with values reported in the literature. No cosolvent was added to minimize the coextraction of undesired compounds and to eliminate the need for subsequent ethanol evaporation, allowing us to proceed directly with further purification steps.^21^

The yield of the extract was 15 ± 1% w/w of the inflorescences, and ^1^H NMR analysis indicated that the CBD concentration increased from the initial 4.5 ± 0.1% w/w in the pristine hemp to 30.8 ± 1.0% w/w. THC concentration also rose from <0.2% w/w to 1.5 ± 0.1% w/w (see Figure S9 in Supporting Information). As widely reported in the literature, this behavior occurs because scCO_2_ extracts all cannabinoids contained in the inflorescences nonselectively.^16^ Significantly, our experiments clearly demonstrated that the extraction process was completed within 3 h, as ^1^H NMR analysis on the hemp residual cake after scCO_2_ extraction showed the absence of CBD (see Figure S10 in Supporting Information). This confirms that we achieved our objective: the complete extraction of CBD from the pristine hemp.^70^ Therefore, no further optimization of the scCO_2_ extraction conditions was deemed necessary.

### 3.3 Winterization Step

ScCO_2_ extraction of hemp inflorescences exhibits selectivity toward the lipophilic fraction composed of terpenes and cannabinoids, as well as volatiles and high molecular weight compounds (i.e., pigments, pheophytins, phospholipids, long alkyl chain fatty acids, etc., generally referred to as waxes), particularly under high-pressure and -temperature conditions.^71^ Their presence complicates the processing of the extract and hinders the thermodynamics of the extraction and isolation of the cannabinoids, implying that they must be removed.^15,21,28,68^ Winterization involves complete dissolution of the extract in a solvent that is usually ethanol^17^ or *n*-hexane,^16^ possibly with heating, followed by fast cooling to −40 °C < *T* < 0 °C to precipitate the waxy fraction, subsequent filtration of the solution, and removal of the solvent.

The procedure we employed in our protocol involved solubilizing the extract in the selected solvent (extract/solvent = 1:3 w/w), heating it to 80 °C for 1 h, followed by rapid cooling to −18 °C for 48 h. The solution was then separated from the waxes, and the solvent was removed under reduced pressure and recovered. The CBD concentration in the dewaxed extract (DE) and in the solid waxy fraction (WF) was subsequently measured by ^1^H NMR. Various winterization solvents (acetonitrile, ethanol, ethyl acetate, and *n*-hexane) were tested, and the results are presented in Table 1.

**Table 1:** Tests for the Winterization of scCO_2_ Extract with Different Solvents^a^

entry	solvent	*m*_DE_ (g)^f^^,^^g^	*m*_WF_ (g)^f^^,^^g^	% CBD (DE)^f^	% CBD (WF)^f^
1	acetonitrile	11.3	8.7	47.7 ± 1.4	9.0 ± 0.4
2^b^	acetonitrile	16.3	3.7	36.0 ± 1.1	8.4 ± 0.3
3	ethanol	12.9	7.1	40.1 ± 1.2	13.8 ± 0.4
4	ethyl acetate	14.0	6.0	36.6 ± 1.1	17.3 ± 0.5
5	*n*-hexane	14.1	5.9	33.1 ± 1.0	26.1 ± 0.8
6^c^	acetonitrile	54.7	45.3	48.5 ± 1.5	9.2 ± 0.3
7^d^	acetonitrile	6.3	39.0	44.5 ± 1.3	6.0 ± 0.2
8^e^	acetonitrile	3.4	35.6	44.3 ± 1.3	2.1 ± 0.1

a Winterization conditions: 20 g of scCO_2_ extract (CBD = 30.8% w/w) was dissolved into 60 g of the selected solvent, and the solution was heated at 80 °C for 1 h. The solution was then rapidly cooled and stored for 48 h at −18 °C. The solution was then filtered, and the solvent was eliminated, yielding a dewaxed extract (DE), while the waxy fraction (WF) composed of waxes and high molecular weight compounds precipitated.

b A solvent/extract = 10:1 ratio was used.

c Same conditions of entry 1 scaled up to 100 g of scCO_2_ extract.

d The WF from entry 5 was redissolved into the appropriate amount of CH_3_CN (extract/solvent = 1:3 w/w), and the winterization step was repeated to recover the residual CBD.

e The WF from entry 6 was redissolved into the appropriate amount of CH_3_CN (extract/solvent = 1:3 w/w), and the winterization step was repeated to recover the residual CBD.

f Each value is a mean of a minimum of two replicated tests.

g The standard deviation around the mean value is ±1.9% of the extract/starting material w/w ratio.

Acetonitrile (entry 1) was the most selective winterization solvent, as indicated by the recovery of the highest amount of waxes (8.7 g). Moreover, CH_3_CN was very selective since the dewaxed extract contained 47.7% w/w of CBD with only 9% w/w of CBD present in the waxy residue. This result was far better than comparable winterization processes reported in literature that led to only 5–15% waxes, hence with a lower purification of the CBD-rich fraction.^28,36^ The other published procedures are usually based on ethanol as a solvent, and the solvent/extract ratio is far higher than the 3:1 ratio selected here, leading to a larger solubilization of undesired substances. Going from a 3:1 to a 10:1 CH_3_CN/extract ratio (entry 2) further supported this hypothesis: a poorer separation was obtained, highlighting that the use of a large excess of solvent leads to the dissolution and solubilization of waxes. Entries 3 and 4 show the results of the winterization step with ethanol and ethyl acetate, respectively. These solvents were less selective compared to acetonitrile, leading to a less dewaxed extract (i.e., higher mass extracted) but also to a lower CBD content. This behavior was even more evident when *n*-hexane was used as a solvent (entry 5), which clearly dissolved waxes, leading to a poor separation of the DE and a similar amount of CBD in the DE and WF. The comparison of entries 3–5 with entry 1 hence confirmed that acetonitrile was the best solvent for dewaxing and purification of the CBND extract.

The winterization step was then repeated on 100 g of scCO_2_ extract with 300 g of CH_3_CN (entry 5), and the results were similar to the ones obtained on the smaller 20 g scale of extract, demonstrating the scalability of the process (m_DE_Extract_ = 65.4 g, 44.2%w/w CBD).^72^

Finally, it is important to note that a small amount of CBD (9.2% w/w, entry 6) remained trapped in the waxy fraction during the winterization step. This phenomenon is documented in the literature, and recent studies stated that 2–30% w/w of the waxy byproduct is made up of CBD and CBDA when a decarboxylation step prior to dewaxing is not performed.^36,45^ To address this issue and to recover the maximum amount of CBD, two consecutive winterization steps were carried out on the WF from entry 6 with a solvent/extract ratio of 3:1. After the third step, almost quantitative extraction of CBD was achieved, yielding a total of 67 g of DE containing 47.8% w/w CBD starting from 100 g of scCO_2_ extract, and only 2.1% w/w of CBD remained trapped in the waxy fraction (entry 8, Table 1).

Although optimal performance is achieved with acetonitrile, its non-GRAS status must be considered. This prompted us to explore also a safer, nontoxic, and renewable alternative such as ethanol. For comparison, three cycles of winterization were conducted on 100 g of scCO_2_ extract by using EtOH as the solvent, and the data are reported in Table S1. The efficiency of ethanol was satisfactory, albeit slightly lower than that of CH_3_CN. Ethanol did not fully extract CBD, with a larger fraction compared to CH_3_CN remaining trapped in the waxy fraction even after the third cycle (*m*_WF_ = 28.7 g, % CBD_WF_ = 7.7% w/w).

It is important to notice that the two best solvents (i.e., CH_3_CN and EtOH) have similar Hildebrand solubility parameters (ethanol: 12.92, acetonitrile: 11.90, ethyl acetate: 9.1, and *n*-hexane: 7.24), while the use of solvents with lower values led to inefficient extraction. The aprotic nature of CH_3_CN probably contributes to its improved performance compared with a protic polar solvent such as ethanol.

### 3.4 C18-Reversed-Phase Silica Filtration

While chromatography is a widely adopted method for purifying cannabinoids, it often involves costly techniques like HPLC, partition chromatography, and two-dimensional LC, with high silica/substrate ratios.^6,21,28,34,35,67,73^ Our goal was to streamline the process by using simple liquid chromatography with a low silica/extract ratio to boost CBD concentration in the extract and enable its selective crystallization, cutting both costs and environmental impact. However, direct chromatography on the winterized extract was not possible due to residual lipophilic compounds contained in it.^15,68^ To overcome this issue, we first filtered the dewaxed extract using C18-reversed-phase silica under vacuum.

Various parameters were investigated to optimize the C18-RP filtration and to improve the selectivity toward CBNDs:

- Silica/extract ratio
- Amount and type of elution solvent
- Solvent for silica regeneration

The filtration process was optimized by selecting the proper solvent, reducing the silica/substrate ratio, and incorporating washing and regeneration steps, enabling the reuse of C18-RP silica. This strategy not only minimized the environmental impact but also significantly reduced both the waste generated and the overall costs associated with the use of C18-RP silica.^73^ Additionally, all solvents were recovered and reused, as previously implemented in the winterization step.

The filtration process was carried out using a glass column filled with C18-reversed-phase (C18-RP) silica. The column was equipped with a porous septum at its base and connected to a vacuum outlet. The dewaxed extract (DE) was dissolved in the minimum required amount of the selected solvent and applied directly to the silica. Filtration proceeded under vacuum using 500 mL of the selected solvent, yielding the first fraction, termed the C18-extract. Upon complete elution of the solvent, the C18-RP silica was subsequently washed and regenerated by the elution of a second solvent, resulting in a second fraction labeled C18-waste. These two fractions (C18-extract and C18-waste) were concentrated by rotary evaporation to determine the weight and the %w/w of CBD in each fraction. Results are summarized in Table 2.

**Table 2:** Tests for the C18-Reversed-Phase Silica Filtration of Dewaxed Extract with Different Solvents^a^

entry	solvent	*m*_C18-extract_^c^^,^^d^	m_C18-waste_^c^^,^^d^	% CBD_C18-extract_^c^
1	acetonitrile	16.1	3.9	59.1 ± 1.8
2	ethanol	18.2	1.8	51.6 ± 1.5
3	ethyl acetate	19.9	0.1	47.2 ± 1.4
4^b^	acetonitrile	15.7	4.3	60.1 ± 1.8

a Experimental conditions: 20 g of the dewaxed extract (47.8% w/w CBD) was solubilized in the minimal amount of the selected solvent (∼10 mL) and placed directly on the stationary phase (C18-RP silica/extract = 3:1 w/w) previously dried-packed into a column. The filtration was performed under vacuum with 300 mL of the selected solvent. The amount of CBD trapped in the C18-waste fraction was always negligible (<0.2% w/w).

b C18-RP silica/extract = 10:1 w/w ratio was used.

c Each value is a mean of a minimum of two replicated tests.

d The standard deviation around the mean value is ±3.5% of the extract/starting material ratio.

As for the winterization step, acetonitrile (entry 1, Table 2) was the solvent of choice for the C18-RP filtration, yielding 16.1 g of C18-extract containing 59.1% w/w CBD. In this step, the absence of CBD from the C18-waste fraction confirmed the complete elution of CBD regardless of the solvent used. With a view of optimizing the amount of solvent for the filtration, fractionation of the eluate into 50 mL aliquots was carried out. Aliquots 3–6 were the only ones containing CBD, indicating that 300 mL was sufficient for the filtration of 20 g of extract. When the filtration was performed with ethanol (entry 2, Table 2), a slightly higher yield of extract was obtained (18.2 g), with a consequently lower CBD concentration. When ethyl acetate was used (entry 3, Table 2), all of the extract was eluted, making the C18-RP filtration step ineffective with this solvent. This prompted us to exploit ethyl acetate as a solvent for silica regeneration.^74^ A test with an increased amount of silica (silica/extract ratio: 10:1, entry 4) showed only a very slight improvement of the separation; hence, we maintained the initial 3:1 silica/extract ratio.

It is important here to note that the C18-RP silica filtration was not feasible directly on the scCO_2_ extract since the nondewaxed extract led to clogging of the silica and difficulty in the elution of the CBNDs. At the same time, this filtration step was crucial to enable the subsequent chromatographic purification.

Lastly, to assess the scalability of C18-RP filtration, a larger column was used with 150 g of extract and the same silica/extract ratio of 3:1. A dry-packed column was utilized, eluting with 2 L of CH_3_CN followed by 1 L of ethyl acetate for silica regeneration. The results (*m*_extract_ = 118 ± 5.2 g, % CBD w/w = 60 ± 1.8%) were consistent with those obtained from the 20 g tests, and the silica could be reused up to 15 times without compromising filtration efficiency.

### 3.5 Chromatographic Purification on Silica Gel

The final step in the CBD purification process involved flash chromatography on silica gel, which posed the most significant environmental challenge since the silica could not be recovered or reused. Despite this, we explored a range of silica/extract ratios between 3:1 and 10:1, identifying that a 7:1 ratio was ideal for efficient purification, balancing both the yield and environmental impact. The optimal mobile phase consisted of petroleum ether/ethyl acetate in a 9:1 ratio. Silica (350 g) was packed with petroleum ether, while the C18-extract (50 g, 60% w/w CBD) was mixed with additional silica and PE to obtain a solid phase that could be easily placed on the stationary phase. The eluent flow was set to 35 mL/min, and 100 mL fractions were collected and analyzed.

After elution of the first four fractions containing the pure mobile phase, the subsequent fractions contained CBD, as indicated by TLC and as summarized in Table 3.

**Table 3:** Flash Chromatography on Silica Gel of the C18-Extract^a^

no. fraction	*m*_extract_ (g)^b^^,^^c^	% CBD (w/w)^b^
1–4	0	
5–10	29.2	82.3 ± 2.5
11–15	5.3	64.5 ± 1.9
16–19	2.5	50.3 ± 1.5
20–30	4.8	34.3 ± 1.0

a Experimental conditions: 50 g of the C18-extract (60% w/w CBD) was solubilized in 20 mL of PE, mixed with 10 g of silica gel, and placed directly on the stationary phase previously wet-packed with PE. The mobile phase was PE/AcOOEt 9:1 v/v. The fractions (100 mL) were controlled by TLC, and the fractions 5–10, 11–15, and 16–19 were separately collected, and the solvent was removed. %w/w was calculated by ^1^H NMR.

b Each value is a mean of a minimum of two replicated tests.

c The standard deviation around the mean value is ±3.7% of the extract/starting material ratio.

It was evident that CBD and THC could not be separated with such a low silica/extract ratio, but the amount of CBD increased to >80% w/w, which was our objective. Fractions 5–15 were collected, and the solvent was evaporated, resulting in an oily extract (34.5 g, 80.6% w/w CBD). This extract was placed in a crystallizer with a few CBD crystallization seeds and stored in the refrigerator at 4 °C. Selective crystallization of pure CBD was observed, provided the content of CBD in the oily extract was >80%. THC remained dissolved in the oily mother liquor. Crystals were washed with cold *n*-hexane in a Büchner funnel and then finally dried under vacuum (40 °C, 0.1 mbar). 3.4 g of CBD was obtained with >99% purity as determined by ^1^H NMR and GC–MS (see Figures S3–S7, S13, and S14). The yield was around 11% of the initial amount of CBD contained in the extract, which was still not completely satisfactory.

Chromatographic purification was scaled up to 500 g of extract, in this case, using gravity elution (see figure S11). With such a high amount of extract, the crystallization of CBD was stunningly improved: 345 g of extract was collected from chromatography. After solvent evaporation, the extract was crystallized as described above to yield 183 g (61% of the CBD contained in the extract) of yellowish CBD crystals. These were redissolved in the minimal amount of hot *n*-hexane and allowed to recrystallize at room temperature, yielding 155 g of pure white CBD crystals and a remarkable 52% yield with respect to the theoretic CBD present initially. It is noteworthy that the mother liquor recovered by this last step was essentially the only fraction of the whole procedure where not-negligible CBD was lost.

A simplified comparison of our process with papers in the literature that aim to the purification of CBD is depicted in Table 4, while a complete comparison of all the protocol steps is reported in Supporting Information (Table S2). The CBD yield obtained by us (entry 7) is significantly higher (52% vs 0.29–15.3, if any), and the purity is comparable or even superior to the other reported ones (entries 1–6, Table 4). It is evident that our protocol is a well-scaled-up process compared to the other scientific reports (600 g vs 1–100 g, entries 1–5, Table 4) apart from the one that provides for the analytical characterization of a commercial CBD extraction sequence of a manufacturer processing 1000 kg day^–1^ of dry hemp inflorescences (entry 6, Table 4). Another advantage of our protocol is the feasibility of the process on CBD-poor inflorescences, while other processes are developed for CBD-rich hemp strains (entries 2 and 6, Table 4). The comparison shown in Table S1 emphasizes the advantages achieved through our protocol in minimizing the loss of CBD at each step of the process. In commercial operations, the content of CBD in distillation residues (waxes, tars, resins, and other byproducts) ranges from 35% up to 81% of cannabinoids (i.e., mostly CBD), which are not recovered since the high temperature required for distillation makes the sticky and harsh residues difficult to treat again, producing three kinds of byproducts that are discarded without any reprocessing to recover CBD.^36^ The procedure proposed in entry 2 took a CBD loss of 5.5%, 1.7%, and 15.7% w/w in the extraction, winterization, and CBD purification steps, respectively.^45^ From Table S1, it is also evident that the CBD/CBDA content in pristine hemp, the CBD loss in each step, the exact solvent/extract ratio, the silica/extract ratio, and the overall CBD yield are often reported ambiguously.

**Table 4:** Comparison of the Process for the Isolation of CBD Starting from Hemp Inflorescences

entry	Hemp Inflorescences		Isolated CBD		reference
	CBD- CBDA (% w/w)	amount extracted (g)	yield (%)	purity (%)	
1	6.2	18	nr	79	(28)
2	18–20	1	nr	91	(45)
3	nr	100	0.63	>98	(40)
4	nr	12	0.29	89.7	(41)
5	3	100	15.3	92.3	(6)
6	17	1 × 10^6^	nr	95–99.5	(36)
7	4.5	600 g	52	>99	This work

Our study demonstrates an easily reproducible and scalable process for the extraction, isolation, and purification of cannabidiol, achieving a 52% yield with a purity exceeding 99%. Additionally, an oily mother liquor is obtained, which can be further processed to recover residual CBD and other cannabinoids.

While decarboxylation and scCO_2_ extraction from pristine hemp have been extensively studied, we focused on optimizing key downstream steps—including winterization, C18-reversed-phase silica filtration, and chromatography—to minimize CBD loss and enhance the recovery and recyclability of materials and solvents, improving the overall efficiency of the process.

Notably, this approach eliminates the need for high-cost instruments such as preparative HPLC, centrifugal partition chromatography systems, or equipment that generates significant CBD waste, such as wiped-film distillers, making our procedure accessible and cost-effective. By minimizing waste, this strategy contributes to a more economically and environmentally sustainable production process. Future studies will explore the recovery of residual CBD from the mother liquor along with other bioactive compounds such as flavonoids, glycosides, alkaloids, and sesquiterpenes retained in the hemp residual cake.

## Supporting Information Available

Additional characterization, NMR spectra, and complete comparison with other scientific reports regarding the isolation of CBD (PDF)
