# Supplementary material for: From Hemp to CBD Crystals: A Scaled-Up Procedure for the Selective Extraction, Isolation, and Purification of Cannabidiol
Source: ACS Agric Sci Technol. 2025 Feb 17;5(3):346–54. doi: 10.1021/acsagscitech.4c00462 (PMC11921148; doi:10.1021/acsagscitech.4c00462)
Supplement: Supplementary file 1 — as4c00462_si_001.pdf [file as4c00462_si_001.pdf]

Supporting info

From hemp to CBD crystals: a complete, efficient and scaled-up procedure for the selective extraction, isolation and purification of cannabidiol

Roberto Calmanti*, Maurizio Selva, Alvise Perosa

Dipartimento di Scienze Molecolari e Nanosistemi, Università Ca' Foscari Venezia, Via Torino 155, 30172, Venezia Mestre (Italy). Email: roberto.calmanti@unive.it

Pilot scale plant

Hemp inflorescences were extract in OL94 GREEN OIL - supercritical CO_2 extractor pilot plant built by Berengo SPA, Via dell'elettricità 2, Marghera-Venezia, Italy. Nominal System dimensions (L x W x H): 2000 x 5000 x 2800

Material description: AISI 316L type containers; AISI 304 type load-bearing carpentry; AISI 316L type pipes and valves; AISI 304 type attacks and connections.

The plant consists of the following units:

**Description of main units**	**Volume (dm^3)**	**Quantity**
extractor	8	1
accumulation tank	20	1
gravimetric separator	4	1
cyclonic separator	2	2
capacitor		1
exchanger		3
CO_2 pump flow rate	45	1
Cosolvent pump flow rate	15	1

The operating conditions and parameters considered in the mechanical sizing of the containers are:

**Maximum process pressure:**	Extractor	350 bar
	1st separator	250 bar
	2nd separator	250 bar
	CO_2 accumulation	150 bar
**Maximum design temperature:**	all units	100 °C
	accumulation, filter	50 °C
**Minimum design temperatures:**	heated units	-10 °C
	accumulation	- 20 °C
**Process temperatures:**	extractor	35 - 85 °C
	1st separator	20 - 50 °C
	2nd separator	30 - 50 °C
	CO_2 accumulation	-20 - 20 °C

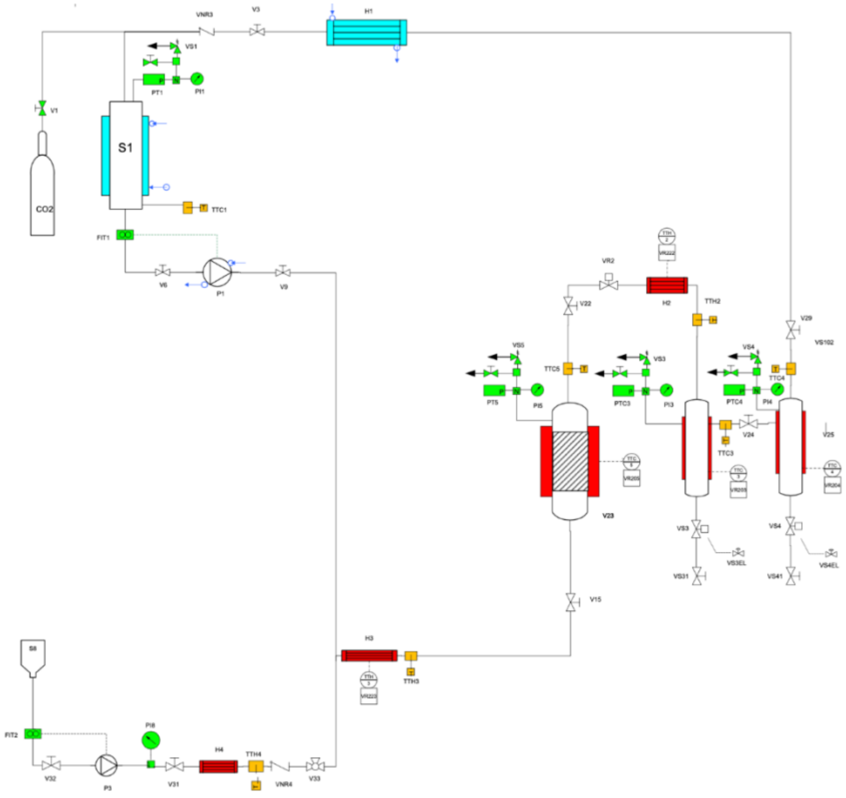

Figure S 1: Schematic representation of the supercritical CO_2 extractor

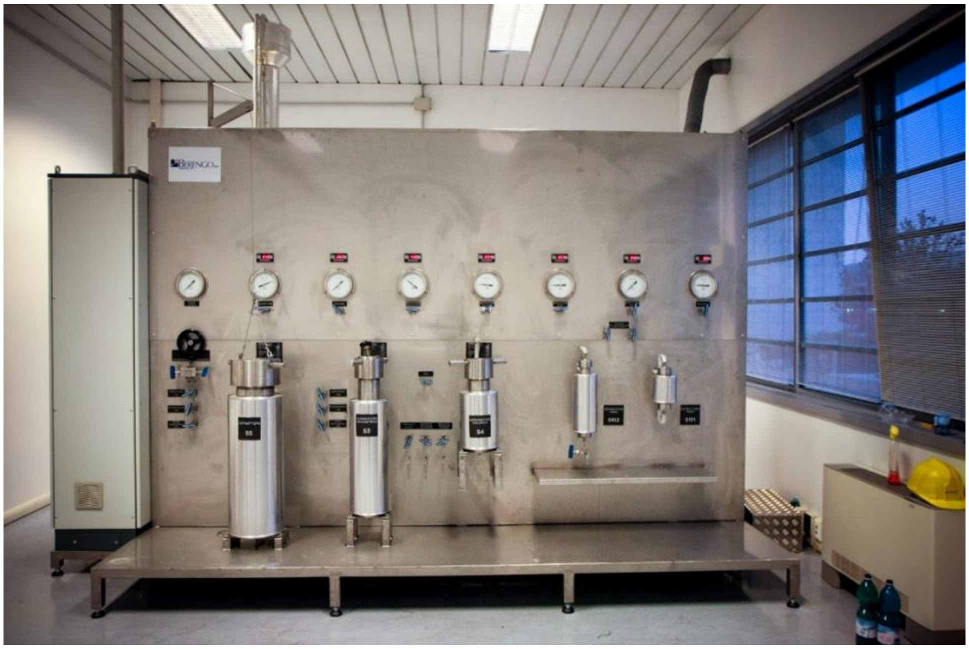

Figure S 2: Picture of the supercritical CO_2 extractor

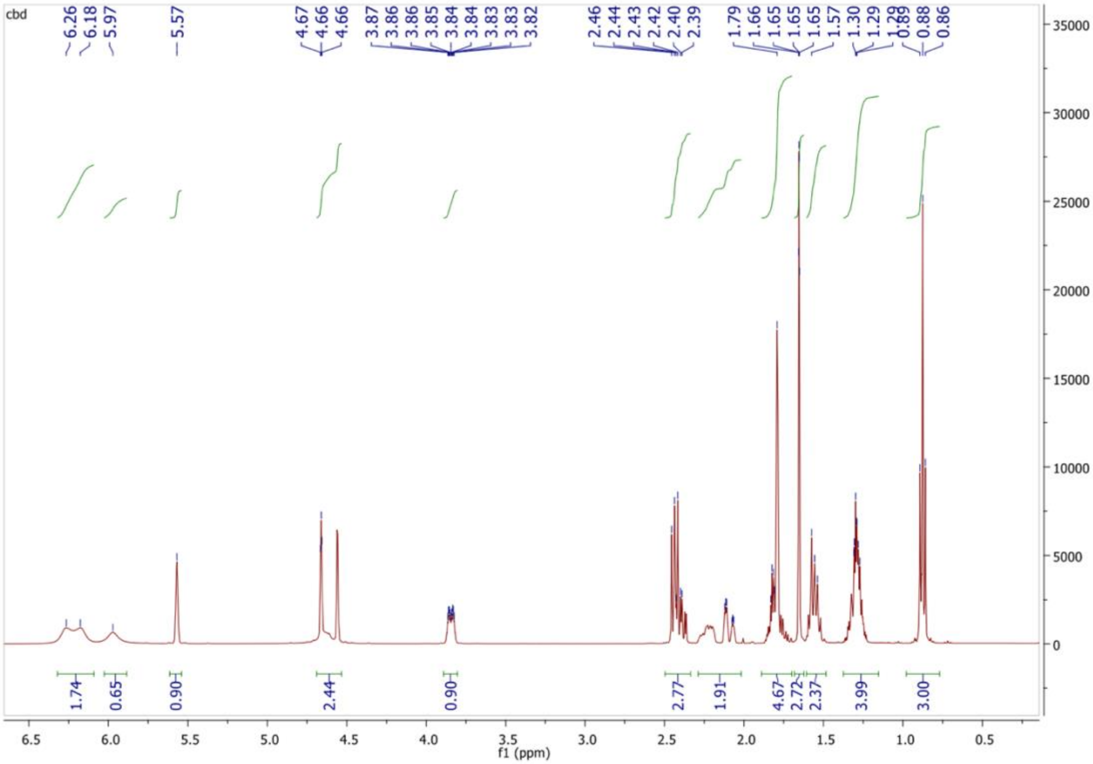

Figure S 3: ^{1}H NMR of Cannabidiol (400 MHz, $CDCl_3$, 298 K) δ (ppm): 0.88 (t, 3H, J = 6.9 Hz), 1.22-1.33 (m, 4H), 1.50-1.60 (m, 2H), 1.64-1.66 (m, 3H), 1.67- 1.79 (m, 5H), 2.04-2.25 (m, 2H), 2.39-2.46 (m, 3H), 3.82- 3.87 (m, 1H), 4.53-4.69 (m, 3H), 5.55-5.59 (m, 1H), 5.97-6.35 (m, 3H).

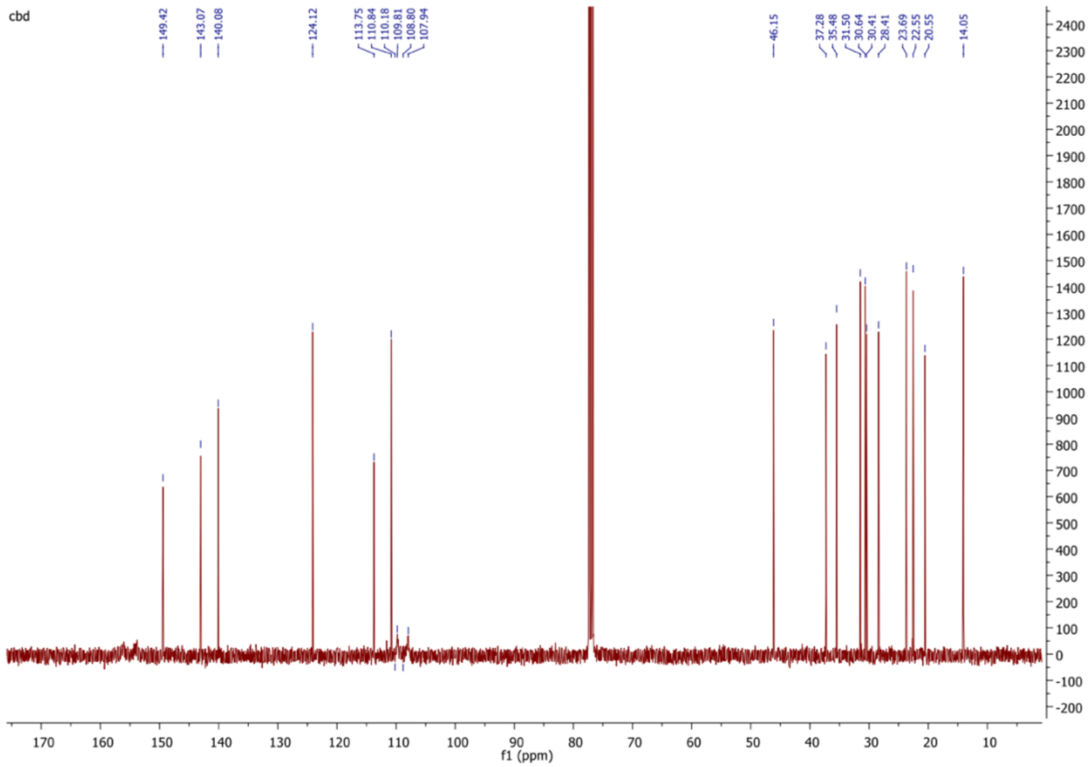

Figure S 4: ^{13}C NMR of CBD (100 MHz, $CDCl_3$, 298 K) δ (ppm): 14.05, 20.55, 22.55, 23.69, 28.41, 30.41, 30.64, 31.50, 35.48, 37.28, 46.15, 107.94, 109.81, 110.84, 113.75, 124.12, 140.08, 143.07, 149.42.

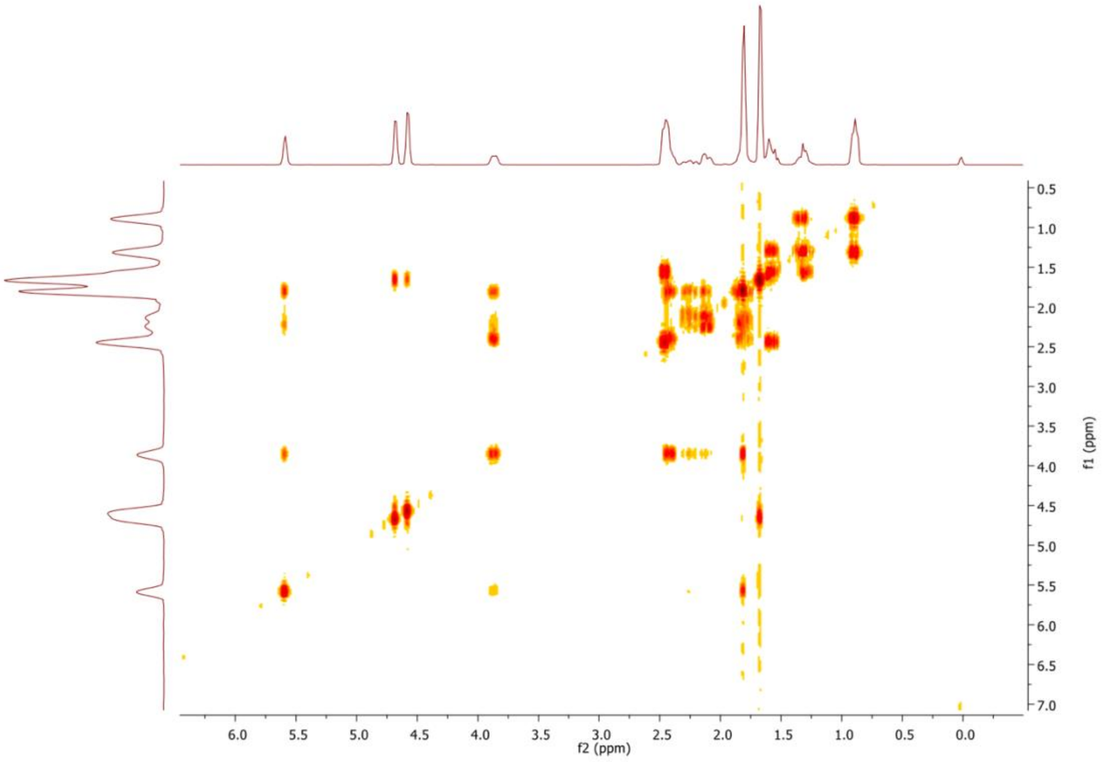

Figure S 5: COSY NMR of CBD

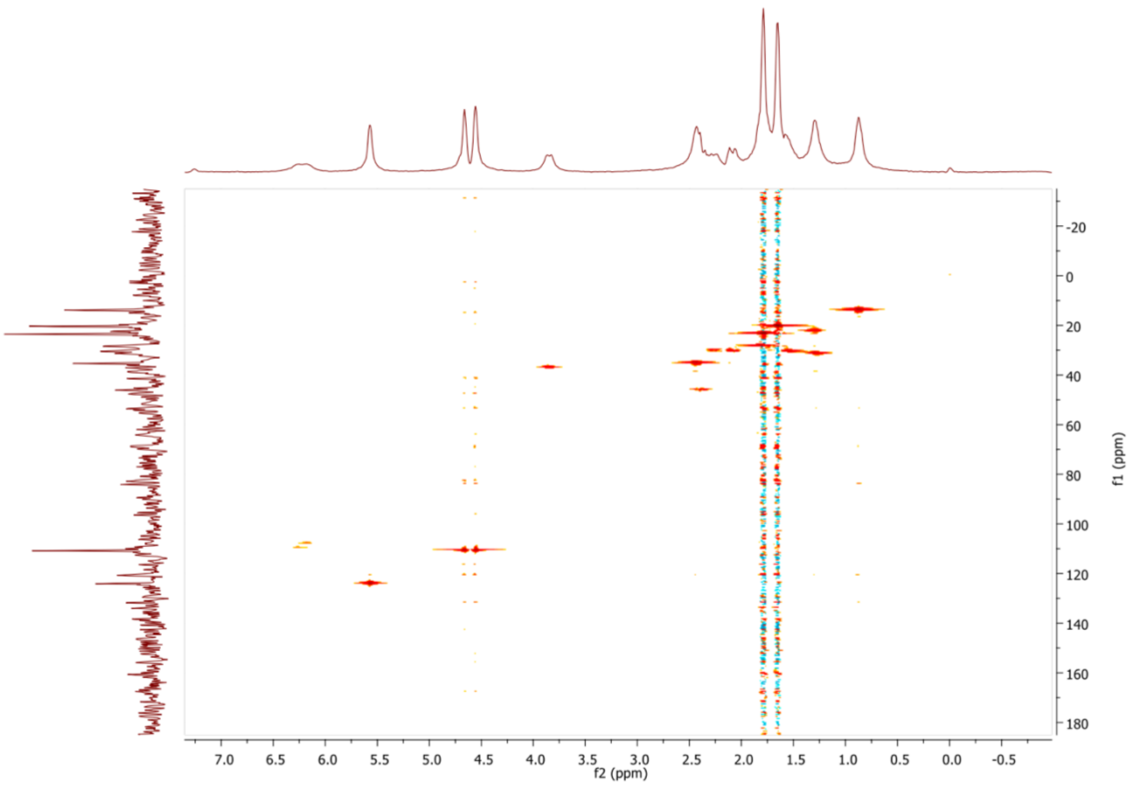

Figure S 6: HMQC of CBD

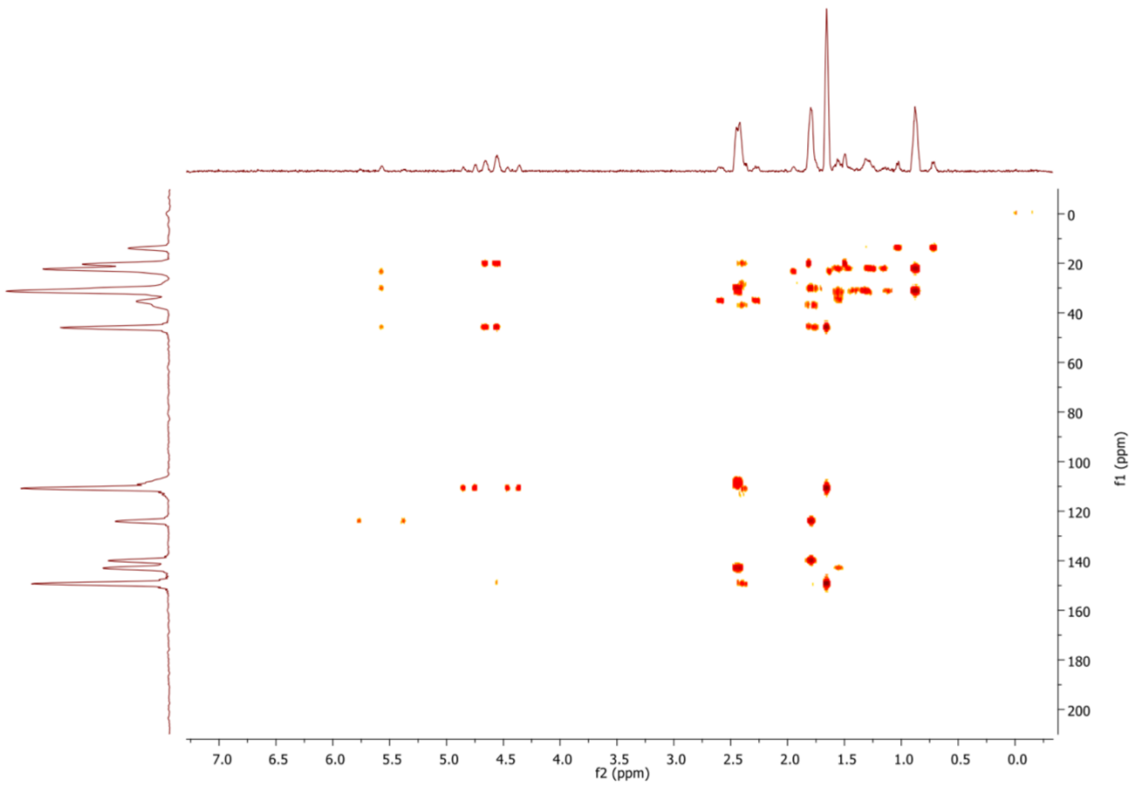

Figure S 7: HMBC of CBD

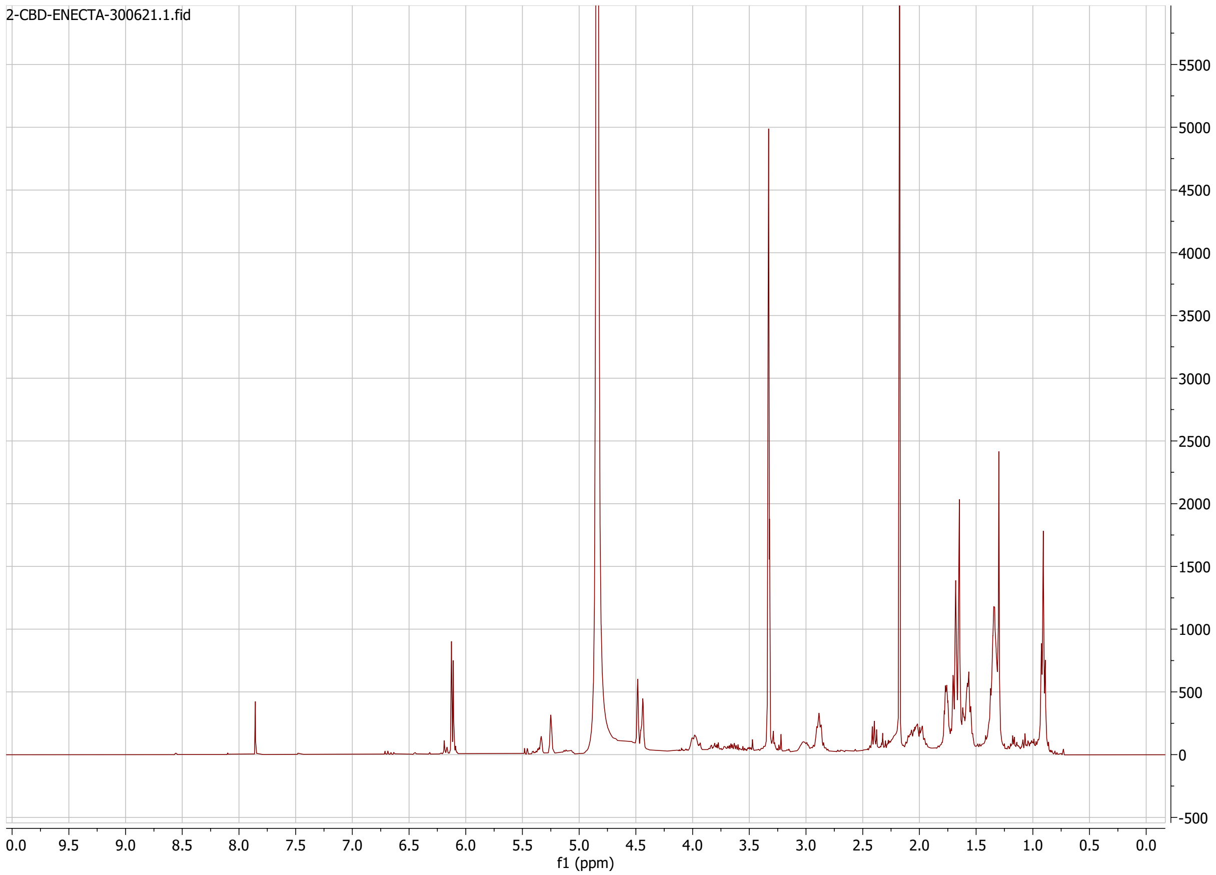

Figure S 8: ^{1}H-NMR spectra of pristine hemp prior to $scCO_2$ extraction

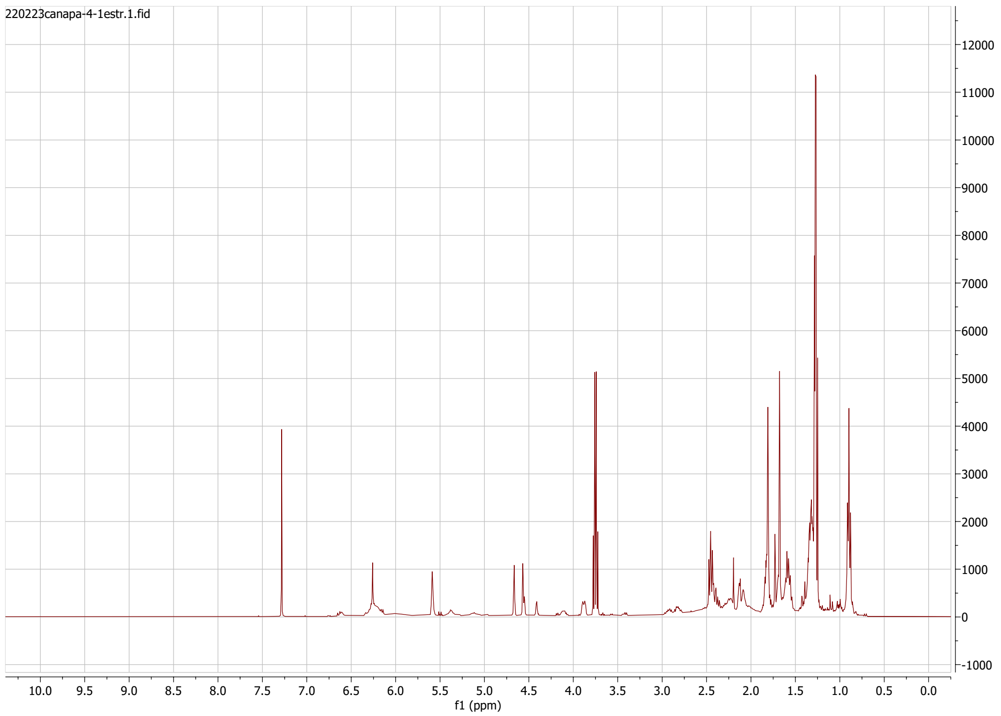

Figure S 9: ^{1}H-NMR spectra of scCO2 extract containing CBD and THC

Table 1: Tests for the winterization of 100 g of scCO$_2$ extract with ethanol. [a]

Entry	Solvent	m_{DE} (g)	m_{WF} (g)	% CBD (DE)	% CBD (WF)
1	CH_3CH_2OH	64.5	35.5	40.7	13.0
2	CH_3CH_2OH	4.8	30.7	36.0	9.4
3	CH_3CH_2OH	2.0	28.7	33.6	7.7

[a] Winterization conditions: 100 g of scCO$_2$ extract (CBD=30.8% w/w) was dissolved into 300 g of ethanol and the solution was heated at 80°C for 1h. The solution was then rapidly cooled and stored for 48h at -18°C. The solution was then filtered, and the solvent eliminated yielding a Dewaxed extract (DE), while the waxy fraction (WF) composed by waxes and high molecular weight compounds precipitated.

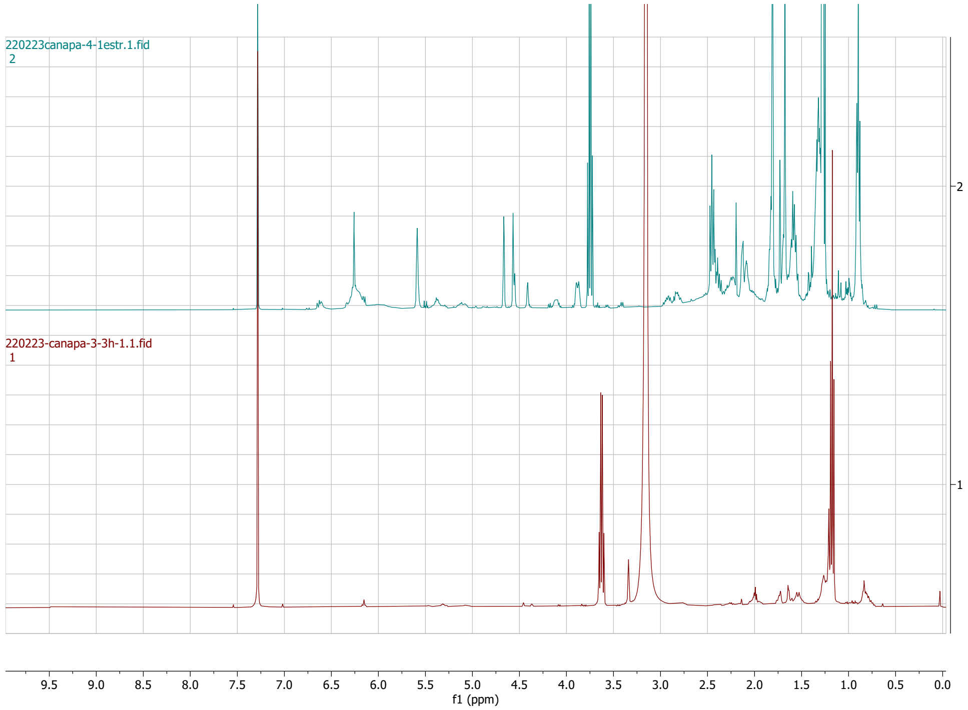

Figure S 10: Comparison of the ^{1}H-NMR spectra of pristine hemp before (green spectra) and after (red spectra.) the scCO$_2$ extraction. The lack of peaks between 6.5 ppm and 4.0 ppm in the red spectra are indicative of the complete extraction of cannabinoids.

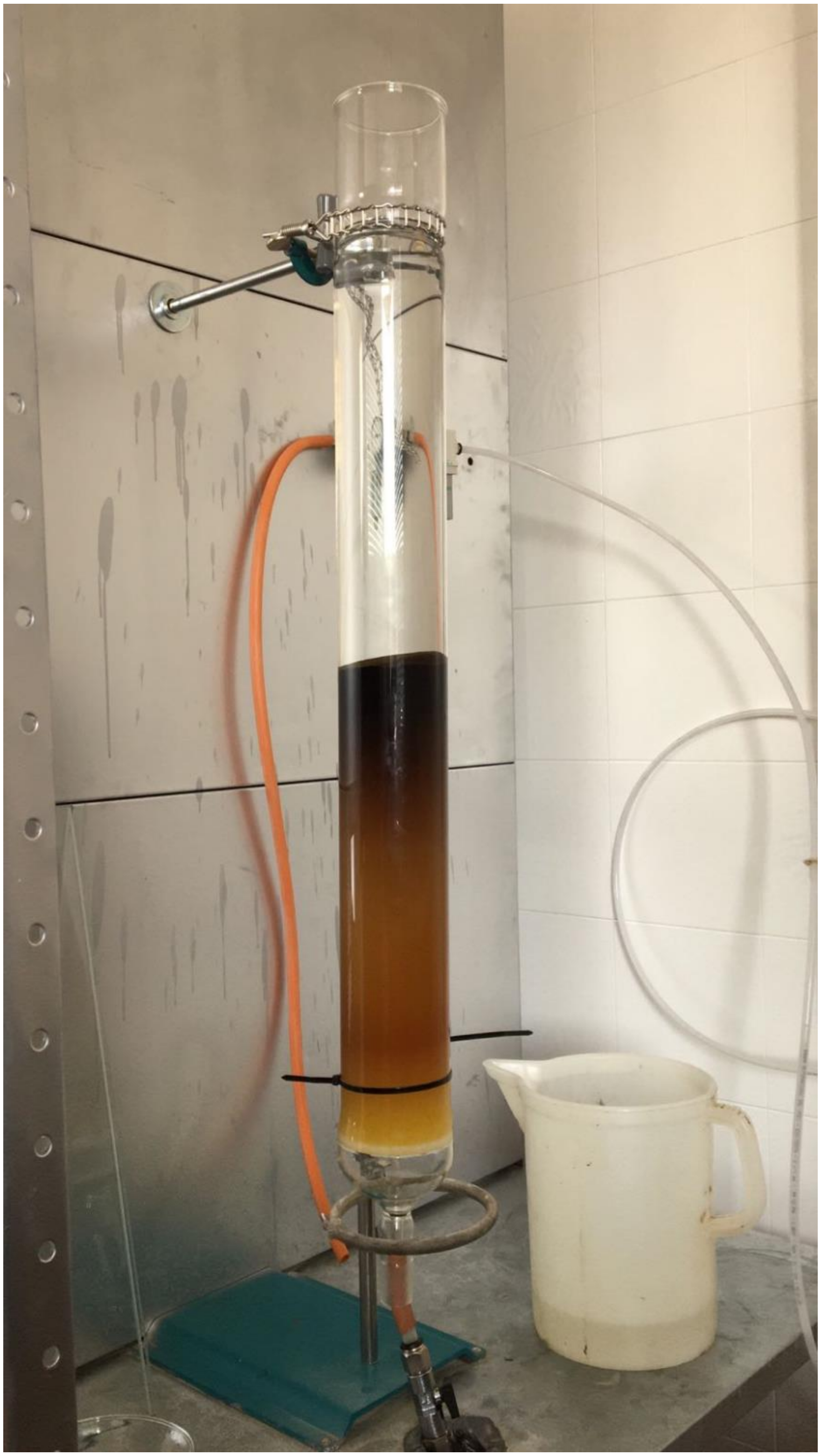

Figure S 11: Scaled up cromatography column for the purification of Cannabidiol

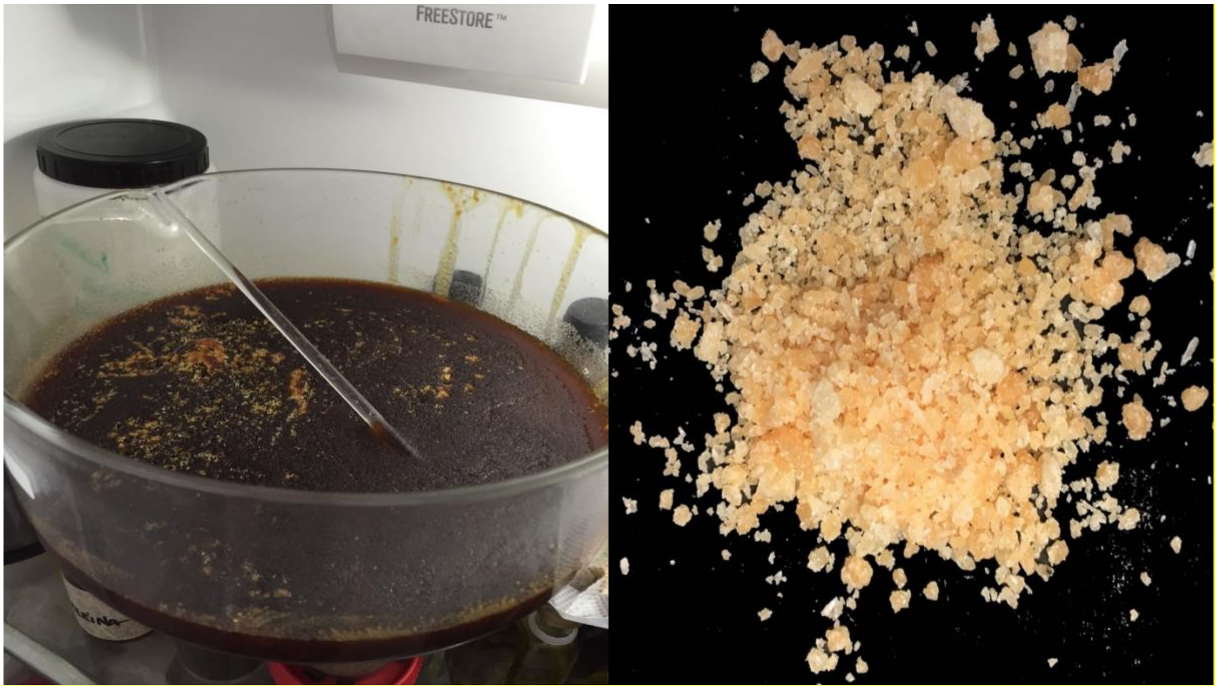

Figure S 12: a) CBD-rich oil obtained after silica gel chromatography; b) CBD crystals obtained after the first crystallization

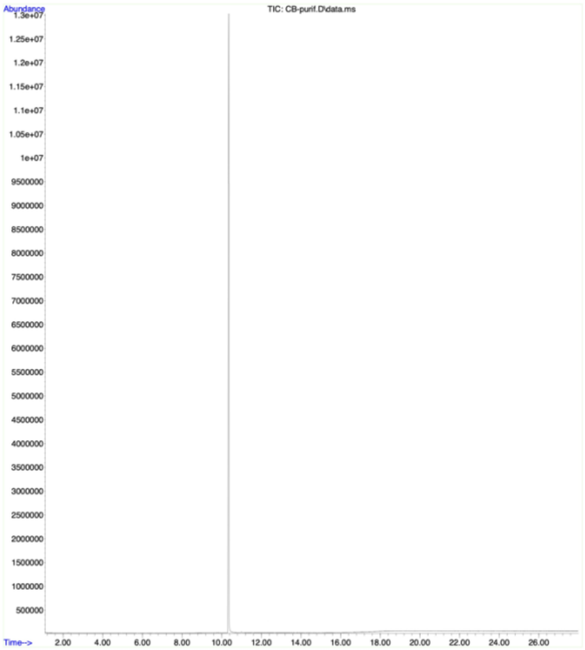

Figure S13: GC chromatogram of the CBD extracted through the complete protocol reported in figure 1

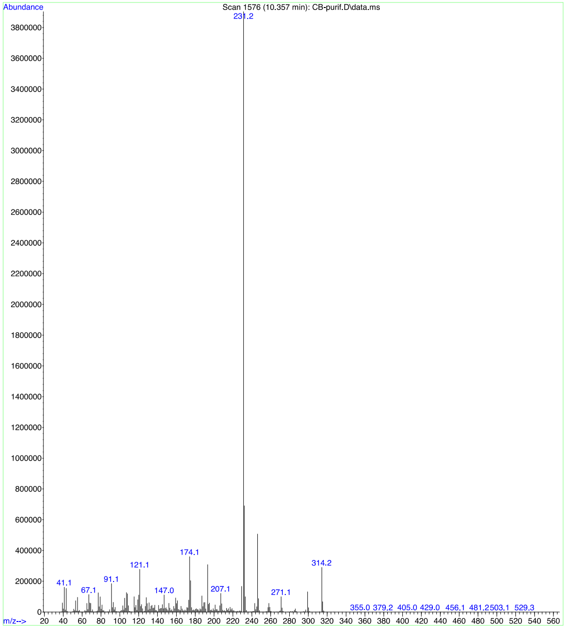

Figure S14: Mass spectrum of cannabidiol

Table S 2: Complete comparison between literature processes for the isolation of CBD from hemp inflorescences

Entry	Hemp inflorescences		Decarboxylation		Extraction		Winterization				CBD purification			
Reference	CBD-CBDA (%w/w)	Amount extracted (g)	Parameters	CBD/CBDA loss (% w/w)	Type, parameters	CBD/CBDA loss (% w/w)	Parameters	CBD/CBDA loss (% w/w)	Type, parameters	CBD/CBDA loss (% w/w)	Type, parameters	CBD/CBDA loss (% w/w)	Yield (%)	CBD purity (%)
1 - (Marzorati et al., 2020)	6,2	18	100°C- 6 h, air	nr	scCO2, 380 bar, 60°C	nr	ethanol; solvent extract ratio = 10 1	nr	c18-RP silica flash cromatography Silica extract ratio: nr	nr			nr	79
2 - (Olejar et al., 2021)	18-20	1	PLE with water 110 bar, 140*C,Olh	0.1	PLE with ethanol 110 bar, 120°C, 0.1 h	5,5	ethanol, solvent extract ratio = nr	1,7	C18-RP silica flash cromatography (40 g), silica:extract ratio = nr	15,7			nr	91
3 - (Appendino et at, 2006)	nr	100	120°C, 2h, air	nr	Acetone x3 solvent:hemp ratio = 3:1	nr	no		Silca gel gravity cromatography silica:extract ratio 6:1	nr			0,63	>98
4 - (Martinenghi et al., 2020)	nr	12	no		ethanol x3 solvent:hemp ratio nr	nr	no		C18-RP silica flash cromatography, silica:extract ratio 175:1	nr			0,29	89,7
5 (Hazekamp, 2007)	3	100	180°C, 0.2 h, air	nr	hexane x3, solvent:hemp ratio 12.5:1	nr	no		Centrifugal partition cromatography hexane:acetone:acetonitrile ratio 5:2:3	nr			15,3	92,3
6 - (Leyva-Gutierrez et al, 2020)	17	$1*10^6$	no		Ethanol solvent:hemp ratio nr	nr	ethanol, solvent:extract ratio nr	35,7	1) Distillation, 22-150°C, pressure < 1mbar	62,2	2) Wiped film distilation, 22-150°C, pressure < 0.4 mbar	80,7	nr	95-99,5
7- this work	4,5	600	80°C, 24 h, air	nr	scCO2 250 bar, 40*C	0	ch3cn x3; solvent:extract ratio 3:1	2,1	I) C18-RP silica filtration silica:extract ratio 3:1	0	2) Silica gel flash cromatography Siica:extract ratio 7:1	0	52%	>99%

Istituto Zooprofilattico Sperimentale del Mezzogiorno
Dipartimento di Chimica - U.O.C. Controllo Merceologico Alimenti O.A.
U.O.S. Controllo Chimico Alimenti Vegetali
Via Salute, 2 80055 Portici (NA) Tel.0817865111 Fax.0817763125

ACCREDIA
L'ENTE ITALIANO DI ACCREDITAMENTO
LAB N° 1043 L
Membro degli Accordi di Mutuo Riconoscimento EA, IAF e ILAC
Signatory of EA, IAF and ILAC Mutual Recognition Agreements

RAPPORTO DI PROVA

Rapporto di Prova N° **217846** **Data Generazione** **02-12-2020**

Campione N° 1 **di Aliquote 1** **Identificativo TED2VD20 / BIOMASSA**

PROVE EFFETTUATE

Prova Determinazione di cannabinoidi in Cannabacee mediante HPLC
Metodo di Prova MP/CH/006 Rev. 5 2020
Data inizio prove 27-11-2020 **Data fine prove** 01-12-2020

	**Risultato**
DELTA 9-TETRAIDROCANNABINOLO (THC)	0.06 g %
TETRAIDROCANNABIVARINA (THCV)	Non rilevabile <0.017 g%
ACIDO CANNABIDIOLICO (CBD-A)	4.00 g %
CANNABIDIOLO (CBD)	0.79 g %
ACIDO CANNABIGEROLICO (CBG-A)	0.05 g %
CANNABIGEROLO (CBG)	Non rilevabile <0.017 g%
CANNABINOLO (CBN)	Non rivelabile < 0.017 g%
DELTA 8-TETRAIDROCANNABINOLO	Non rivelabile < 0.017 g%
ACIDO DELTA9-TETRAIDROCANNABINOLICO A (THC-A)	0.11 g %

Il Dirigente Responsabile delle prove

Rapporto di Prova firmato digitalmente e conforme ai sensi dell'art. 23 DLgs 7 marzo 2005 n°82 CAD e successive modifiche ed integrazioni
Il campionamento è stato effettuato dal cliente/utente.
Il presente Rapporto di Prova riguarda solo i campioni sottoposti a prova e non può essere riprodotto parzialmente salvo approvazione scritta dell'Istituto Zooprofilattico Sperimentale del Mezzogiorno. Per eventuali chiarimenti sul presente Rapporto di Prova contattare il n. tel. 0 8 1 - 7 8 6 5 2 1 4 Per eventuali reclami sul presente Rapporto di Prova contattare il n. tel. 081 7865274 - Ufficio per le Relazioni con il Pubblico

Descrizione prodotto:	CRISTALLI CBD	Lotto.	135

Analisi n.	S5 fermo	Data analisi	24/03/2021
Campione analizzato	CRISTALLI CBD - C2039042		
Data Campione	DDT 235 del 18/03/2021		

Risultati Analisi

Analita	Esito	Metodo
THC	0.00 %	*Estrazione: metanolo*<br>*Determinazione: HR-GC*
CBN	0.16 %	
CBD	99.90 %	

HR-GC: gascromatografo con rivelatore di fiamma.

Macerata, 01/04/21

l Responsabile del Laboratorio

	**FOR.MED.LAB.**<br>***FORENSIC MEDICINE AND LABORATORY S.R.L.***<br>Sede Legale: Via Don Minzoni n. 9 – 62100 Macerata<br>Sede operativa: Via Velluti,38 Fr.ne Piediripa 62100 Macerata<br>Partita IVA/Cod.Fiscale 01952180436<br>Tel. 3669641562 Fax 0733/1876435<br>e-mail: for.med.lab.srl@gmail.com PEC for.med.lab.srl@pec.it	*SPIN OFF* uniMC UNIVERSITÀ DI MACERATA<br>l'umanesimo che innova

Spettabile

ECOHEMP SRL

Viale della Tecnica, 30
45020 - Villanova del Ghebbo (RO)
P.Iva 048056502

DOSAGGIO DI CANNABINOIDI

Descrizione prodotto: **CRISTALLI CBD** **Lotto.** **135**

Analisi n.	S5 fermo	Data analisi	24/03/2021
Campione analizzato	CRISTALLI CBD - C2039042		
Data Campione	DDT 235 del 18/03/2021		

Risultati Analisi

Analita	Esito	Metodo
THC	0.00 %	*Estrazione: metanolo*<br>*Determinazione: HR-GC*
CBN	0.16 %	
CBD	99.90 %	

HR-GC: gascromatografo con rivelatore di fiamma.

Macerata, 01/04/21

l Responsabile del Laboratorio
